# Incidence of Lyme borreliosis following *Ixodes ricinus* tick bites in Poland: a citizen science approach

**DOI:** 10.1186/s13071-025-07133-y

**Published:** 2025-11-28

**Authors:** Julia Koczwarska, Agnieszka Pawełczyk, Renata Welc-Falęciak

**Affiliations:** 1 Department of Parasitology, Faculty of Biology, University of Warsaw, Miecznikowa 1, 02-096 Warsaw, Poland; 2 Department of Immunopathology of Infectious and Parasitic Diseases, Medical University of Warsaw, Pawińskiego 3C, 02-106 Warsaw, Poland

**Keywords:** Lyme borreliosis, Risk of infection, *Ixodes ricinus*, Ticks, *Borrelia burgdorferi* s.l., *Borrelia miyamotoi*, *Erythema migrans*

## Abstract

**Background:**

The risk of developing Lyme borreliosis (LB) following a tick bite is influenced by several independent factors, including the promptness of tick removal, host immune response, and regional variability in pathogen prevalence. This study employed a citizen science approach to investigate the incidence of LB following *Ixodes ricinus* bites in Poland and to assess the relationship between *Borrelia* spirochete load, tick attachment duration, and subsequent LB development in humans.

**Methods:**

The study was conducted over 2 years (2021–2022). Participants were instructed to submit removed ticks and to complete questionnaires at enrollment and 8 weeks post-bite. All LB cases were physician-confirmed on the basis of national clinical criteria. Tick attachment duration was estimated using scutal and coxal indices. Genomic DNA extracted from ticks was subjected to molecular screening for *Borrelia* spp., and spirochete load was quantified using droplet digital polymerase chain reaction (PCR).

**Results:**

The prevalence of *Borrelia* infection in *I. ricinus* ticks was 15.7% (*n* = 2079). The overall risk of developing LB following a tick bite was 3.1% (*n* = 1757). Among confirmed LB cases (*n* = 54), *erythema migrans* was reported in 64.9%. In cases involving *Borrelia*-positive ticks (*n* = 250), the risk of LB increased with attachment duration—from 10.0% for ticks removed within 24 h to 30.0% for those removed after 48 h. Among *Borrelia*-infected ticks removed from the skin of the patients with LB, *Borrelia afzelii* was the most frequently detected species, however co-infection with *Borrelia miyamotoi* was also observed. Notably, both *Borrelia* prevalence and spirochete load in ticks decreased significantly with prolonged attachment duration.

**Conclusions:**

The overall risk of LB following an *I. ricinus* bite was relatively low. While *B. afzelii* was the dominant species detected, the potential risk posed by *B. miyamotoi* warrants attention, given its significantly higher spirochete load compared with the *B. burgdorferi* sensu lato complex, potentially indicating greater transmission efficiency. The observed decline in spirochete load with increasing attachment time suggests a complex dynamic that may influence transmission risk, meriting further investigation. This study highlights the utility of citizen science as a viable method for collecting large-scale data on human–tick encounters, despite methodological constraints inherent in volunteer-based research.

**Graphical Abstract:**

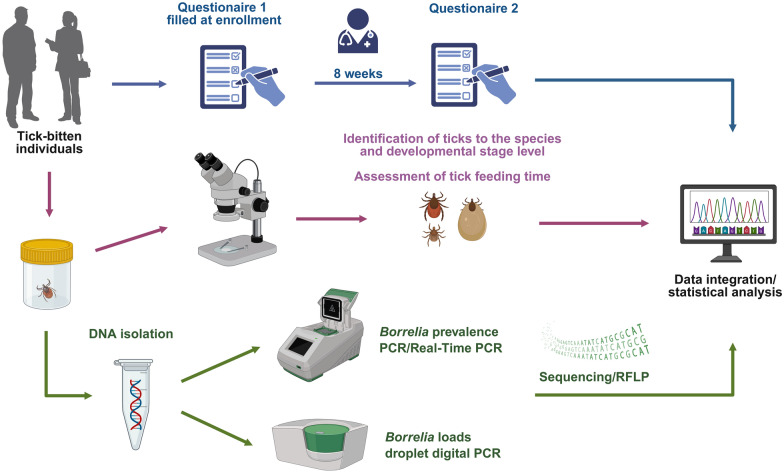

**Supplementary Information:**

The online version contains supplementary material available at 10.1186/s13071-025-07133-y.

## Background

Lyme borreliosis (LB) is one of the most prevalent and well-characterized tick-borne diseases in Europe, with a steadily increasing incidence over the past two decades [1, 2]. In Poland, which is considered an endemic region for ticks and tick-borne infections and where LB has been subject to mandatory notification and registration since 1996, the incidence of LB increased significantly from 4.79 cases per 100,000 population in 2000 to 66.96 cases per 100,000 in 2023 [3]. The most frequently reported clinical manifestations of LB in Poland are *erythema migrans* (EM) and Lyme arthritis (LA), accounting for approximately 74% and 32% of all reported cases, respectively [4]. Diagnosing LB remains challenging due to the non-specific nature of its clinical presentation—particularly in the early stages of infection, before a measurable serological response has developed. Additional complicating factors include the persistence of *Borrelia*-specific IgM and/or IgG antibodies and the relatively high background seroprevalence in certain populations, which can exceed 50% among hunters in endemic areas [5]. Moreover, it has been suggested that LB incidence rates in both Europe and North America may be overestimated. Contributing factors include unnecessary testing, misinterpretation of serological results, and diagnoses based solely on serological findings without meeting clinical case definitions [6–8].

The risk of developing LB following a tick bite depends on multiple spatiotemporally variable factors, including, but not limited to, the tick’s developmental stage and species, the prevalence of *Borrelia* spp. in the tick population, tick density, the presence and abundance of competent reservoir hosts (e.g., rodents and birds), the duration of tick attachment, and individual host susceptibility to infection [2]. In Europe, *Ixodes ricinus* is undoubtedly the primary vector of *B. burgdorferi* s.l. and accounts for approximately 90–100% of all ticks removed from humans [9, 10]. Among its life stages, nymphs are most frequently implicated in the transmission of zoonotic pathogens due to their small size and high feeding activity [9–11]. At least five *Borrelia* species—*B. burgdorferi* sensu stricto (s.s.), *B. garinii*, *B. afzelii*, *B. spielmanii*, and *B. bavariensis*—are recognized human pathogens, some of which may be associated with distinct clinical manifestations [2]. Although *B. lusitaniae* and *B. valaisiana* have occasionally been isolated from human patients, their pathogenic potential is considered limited [2, 12]. *Borrelia miyamotoi*, a recently identified tick-borne pathogen, is phylogenetically more closely related to the relapsing fever group of *Borrelia* than to the LB complex. Its capacity to cause human disease has been confirmed in Europe, North America, and Asia, and *B. miyamotoi* disease (BMD) is now regarded as an emerging public health concern (reviewed in [13]).

In Europe, the estimated risk of developing LB following a tick bite is approximately 2–3%, while asymptomatic seroconversion occurs in about 3–5% of cases [14–21]. Poland is considered a high-risk area for LB in Europe, characterized by substantial geographical variation in incidence and a relatively high *Borrelia* spp. prevalence in *I. ricinus* ticks—reported to be around 20% [7]. However, data on the actual transmission rate of *Borrelia* from ticks to humans—and the associated risk of developing LB following a bite from either infected or uninfected *I. ricinus* ticks in Central Europe, including Poland—remain limited. Although several studies from other European countries have explored this topic [14–17, 19, 20, 22], comparable data for Poland are scarce.

In our study, we employed a citizen science approach, an effective tool in epidemiological research [23, 24], to investigate the incidence of LB following *I. ricinus* bites as well as the influence of *Borrelia* load in ticks and duration of tick attachment on the likelihood of disease development in humans in Poland. Participants who consented to take part in the study and had experienced a tick bite completed two questionnaires: one at the time of enrollment and another 8 weeks after tick submission. Ticks removed from the skin were analyzed using molecular techniques, including real-time polymerase chain reaction (PCR), conventional PCR, and droplet digital PCR. We believe that assessing the risk of LB development following an *I. ricinus* bite may be valuable in clinical decision-making—particularly in identifying individuals at high risk and guiding the consideration of prophylactic antibiotic therapy after tick exposure.

## Methods

### Study design and questionnaires

The study design has been described in detail previously [25]. Briefly, the research was conducted over a 2-year period, from 2021 to 2022. Information regarding the study was disseminated via the University of Warsaw website, medical and healthcare-related online platforms, social media networks, and internal university communication channels, including email outreach to researchers, students, and administrative staff. Participants were instructed to deliver or mail their removed tick(s) to the Department of Parasitology, University of Warsaw, in a securely sealed container filled with 70% ethanol, within five days following tick removal by a healthcare professional or by the individuals themselves. Inclusion in the study was contingent upon obtaining written informed consent. Participants were provided with detailed information regarding the study objectives and procedures. For minors (< 18 years of age), consent was obtained from a parent or legal guardian.

At the time of enrollment, study participants were requested to complete an online questionnaire that collected data on various parameters, including the number of tick bites, the environment in which the tick(s) were encountered (urban versus rural), the anatomical location of tick attachment, and the use of chemoprophylaxis and/or personal protective clothing. All participants were subsequently contacted for an 8-week follow-up via email or telephone. The follow-up questionnaire gathered information on any additional tick bites sustained during the interval, overall health status over the preceding 2 months, and the presence of symptoms potentially indicative of tick-borne diseases, including *erythema migrans* (as confirmed by a general practitioner or infectious diseases specialist). Participants were also asked to provide medical documentation if they had consulted a physician for symptoms potentially associated with LB, as well as details on the results of any serological testing for LB and any antibiotic therapy administered within the previous 8 weeks specifically for suspected LB.

Inclusion in the study was limited to individuals who experienced tick bites, submitted the removed tick(s) to the Department of Parasitology, and completed at least the initial online questionnaire. Participants were permitted to submit multiple ticks only if all ticks had been removed on the same calendar day. The number of responses analyzed varied between questions, as completion of all questionnaire items was not mandatory.

### Exclusion criteria

The individuals who reported a second bite within the 8 weeks from first notification, the patients with immunosuppression and the individuals who declared tick-bite outside Poland were excluded from the study.

### Case definition

The criteria for LB diagnosis in this study were consistent with the recommendations of the Polish Society of Epidemiologists and Infectious Disease Physicians [26]. All reported LB cases were confirmed by physicians on the basis of national clinical guidelines, which included typical clinical manifestations and, where applicable, laboratory testing such as ELISA followed by confirmatory western blot. Serological test results alone, in the absence of clinical symptoms, were not considered sufficient for LB diagnosis. Only physician-confirmed LB cases were included in the analysis.

### Tick collection and identification

Tick specimens removed from humans were collected across various regions of Poland from April to November in both 2021 and 2022. Morphological identification of ticks to the species and developmental stage was performed using a standard taxonomic key [27]. Specimens that were too severely damaged during removal from the skin to permit reliable species-level identification were excluded from the analysis.

### Time of tick feeding

The time of tick feeding on the individuals was estimated only for non-damaged nymphs and females based on the coxal index and additionally on scutal indices in the case of heavily engorged ticks according to Gray et al. [28]. The scutal index was obtained by calculating the ratio of the maximum width of the scutum to the length of the idiosoma, whereas coxal index was determined from the scutal width and the gap between the fourth pair of legs across the ventral abdomen of the tick.

### DNA extraction, real-time PCR and RFLP-PCR analysis

Individual ticks were washed in 70% sterile ethanol and then in sterile water to avoid DNA contamination and then homogenized using sterile, stainless steel beads and automatic TissueLyser II (Qiagen, Germany). Genomic DNA from tick homogenate (180 µl) was isolated using the DNeasy Blood & Tissue Kit (Qiagen, Germany) according to the manufacturer’s protocol. Genomic DNA was used for molecular screening for *Borrelia* spirochetes (real-time PCR *Borrelia* kit) according to the manufacturer’s protocol (EURx, Poland). All positive samples were double-checked using nested PCR through amplification of the flagellin gene (*flaB*) [29]. Restriction fragment length polymorphism (RFLP; [30]) and/or sequencing amplicons in both directions (Eurofins Polska Sp. z o. o.) were used to differentiate *Borrelia-positive* isolates at the species level. Sequenced *Borrelia* DNA obtained from infected ticks was used as positive controls. Obtained nucleotide sequences were analysed using BLAST NCBI and MEGA v. 11.0 software for sequence alignment and species typing using sequences deposited in GenBank NCBI. The new nucleotide sequences have been deposited in the GenBank database under accession numbers PV173335–PV173337, PV173339–PV173341.

### Droplet-digital PCR (ddPCR)

Tick extracts were quantified prior to ddPCR analysis using the NanoDrop One™ (Thermo Fisher Scientific, Waltham, MA) to determine the total concentration of double-stranded DNA present in each sample. The 50 ng of template DNA of the *Borrelia-positive* sample was used in each ddPCR reaction. Samples for which a concentration of 50 ng DNA per reaction could not be obtained due to low or high total concentration of DNA were added at the maximum possible volume or diluted 10 times respectively. Afterwards obtained results were recalculated for the concentration of 50 ng per reaction. Primers p16Swt-fwd and p16Swt-rev were used to amplify the 139-bp fragment of the gene encoding the *Borrelia* 16S rRNA [30]. ddPCR assay was performed in 22 µL reaction mixtures containing 11 µL of QX200^™^ ddPCR^™^ EvaGreen Supermix (Bio-Rad, Hercules, CA), forward and reverse primers at 100 nM each, 50 ng of template DNA and RNase-/DNase-free water on amount depending on the volume of the template DNA. Negative controls were performed in the absence of template DNA. Sequenced *Borrelia* DNA obtained from infected ticks was used as positive control. 20 µL of ddPCR reaction mixture was loaded into an eight-well DG8™ Cartridge (Bio-Rad) and droplets were formed with the Bio-Rad QX200™ Droplet Generator, following the manufacturer’s instructions. Generated droplets were then transferred to a 96-well plate and sealed with a Bio-Rad PX1^™^ PCR Plate Sealer, as recommended by the manufacturer. *Borrelia* 16S rRNA gene fragment was amplified in a C1000 Touch^™^ Thermal Cycler using the following cycling conditions: an initial denaturation step at 95 °C for 5 min, followed by 40 cycles consisting of denaturation at 95 °C for 30 s and an annealing/extension step at 60 °C for 1 min, followed by signal stabilization steps at 4 °C and 90 °C for 5 min each and a 4 °C indefinite hold. The overall ramp rate was set at 2 °C/sec. After cycling, droplets were immediately analyzed on the QX200^™^ Droplet Reader. Bio-Rad QuantaSoft Analysis Pro software was utilized for absolute quantification to determine the concentration of target DNA copies in a sample (copies of template per μL of the final 1 × ddPCR reaction).

### Statistical analysis

Statistical analyses were conducted using IBM SPSS Statistics, version 29.0 (IBM Corp., Armonk, NY, USA). The prevalence of *Borrelia* infection in ticks—defined as the proportion of infected individuals—was estimated using maximum likelihood methods based on log-linear analysis of contingency tables (HILOGLINEAR procedure). This method was chosen due to the categorical nature of both dependent and independent variables and its suitability for modelling multi-dimensional frequency data. Tick infection status was treated as a binary variable (infected = 1, uninfected = 0) and analyzed in relation to year of collection (2021–2022), tick developmental stage (larva, nymph, adult), and estimated feeding duration (< 24 h, 24–48 h, > 48 h). To examine associations between the presence of *Borrelia* DNA in ticks and patient-reported clinical outcomes—both at baseline and at the 2-month follow-up—such as the occurrence of *erythema migrans* (EM) and clinically confirmed LB (diagnosed by a general practitioner or infectious diseases specialist), the same statistical framework was applied. All clinical outcomes were treated as binary variables (present = 1, absent = 0). A complete case analysis approach was employed, excluding any observations with missing data on key variables. No imputation procedures were used.

Quantitative data on *Borrelia* DNA load—measured as the number of target gene copies per μL in the final 1 × droplet digital PCR (ddPCR) reaction—were compared across groups using non-parametric tests: the Mann–Whitney U test for two-group comparisons and the Kruskal–Wallis test for comparisons involving more than two groups. The use of nonparametric methods was warranted by the non-normal distribution of the data, as determined using the Shapiro–Wilk test. All statistical tests were two-tailed, and a *p*-value < 0.05 was considered indicative of statistical significance.

## Results

### Description of the *Ixodes ricinus* bitten participants

Data on participant characteristics—including sex, age, and relevant medical information (e.g., presence of EM, LB diagnosis, laboratory testing, and antibiotic treatment)—were collected and analyzed based on self-reported questionnaires completed at enrollment (*n* = 1956) and at 8 week follow-up (*n* = 1764), corresponding to a response rate of 90.2%. A higher proportion of participants were female (53.9%; 1055/1956) compared with male (46.1%; 901/1956). The mean age of participants was 40 years, with an age range spanning from 1 to 88 years. 64 out of 1956 participants (3.3%) submitted more than one tick, all of which had been removed from the skin on the same calendar day.

### ***Ixodes ricinus*** ticks

A total of 2153 ticks removed by participants were available for further analysis, of which 2079 (97%) were identified as *I. ricinus* and 34 (1.6%) as *Dermacentor reticulatus*; forty ticks could not be identified as species due to being extensively damaged (1.4%). The results of our study on pathogen prevalence in *D. reticulatus* ticks feeding on human skin, as well as the clinical manifestations of tick-borne infections following tick bites, have been published previously [25]. Almost 60% (1239/2153) of all *I. ricinus* ticks were collected in 2022. Of the feeding *I. ricinus*, 89 (4.3%) were larvae, 1,451 (69.8%) were nymphs, and 539 (25.9%) were adults where females were in the vast majority (95%; 510/539). Significant differences in the time of attachment were observed between nymphs and females (*χ*^2^_2_ = 40.02; *p* = 0.0001; Table 1). Most of all females were collected during the first 24 h of attachment (37.2%). Nymphs were removed mainly between 24 and 48 h of attachment (50.0%), whereas only 29.3% of them were recorded during the first 24 h of attachment.

**Table 1:** Percentage of *I. ricinus* nymphs and females removed from human skin (A), *Borrelia* prevalence in *Ixodes ricinus* ticks removed from human skin (B), and LB and EM incidence after *I. ricinus* tick bite (C) depending on the duration of tick attachment

		Duration of tick (nymphs and females) attachment^*^			*P* value
		< 24 h	24–48 h	> 48 h	
A	Number of collected ticks				
Tick stadium					
* Nymphs*	1186	29.3 [26.7–31.9]	50.0 [47.2–52.8]	20.7 [18.5–23.1]	0.0001
* Females*	390	37.2 [32.5–42.1]	32.1 [27.6–36.8]	30.8 [26.3–35.5]	
* Ʃ*	*1576*				
B	Number of tested ticks				
* Borrelia* prevalence	1700	21.6 [18.3–25.1]	15.7 [13.2–18.4]	11.9 [8.9–15.4]	0.0001
C	Number of participants				
Incidence of LB after *I. ricinus* tick bite (infected and non-infected)	1438	2.8 [1.6–4.7]	3.3 [2.1–4.9]	5.6 [3.5–8.4]	0.125
Incidence of LB after infected *I. ricinus* tick bite	250	10.0 [5.3–17.0]	18 [11.4–26.4]	30 [18.7–43.6]	0.010
Incidence of EM after *I. ricinus* tick bite (infected and non-infected)	1438	1.3 [0.5–2.7]	2.4 [1.4–3.8]	3.8 [2.1–6.2]	0.075
Incidence of EM after infected *I. ricinus* tick bite	250	5.0 [1.9–10.6]	12.0 [6.7–19.4]	22.0 [12.3–34.8]	0.008

^*^Values are given as the percentage with the 95% confidence interval (Cl) in parentheses

### *Borrelia* prevalence in *Ixodes ricinus* ticks

Overall, the *Borrelia* infection prevalence in the human-derived *I. ricinus* ticks determined by real-time PCR was 15.7% (326/2079). Statistical analysis of the 2 year period revealed a significant decrease in *Borrelia* prevalence between 2021 (168/840, 20.0%; 95% Cl 17.4–22.8%) and 2022 (158/1239, 12.8%; 95% Cl 11.0–14.7%) (*χ*^2^_1_ = 18.0; *p* = 0.0001). Furthermore, significant differences in *Borrelia* prevalence were observed between different tick stages (*χ*^2^_2_ = 22.2; *p* = 0.0001). *Borrelia* DNA was detected in 3.4% (3/89; 95% Cl 1.0–8.7%) of larvae, 14.7% (213/1451; 95% Cl 12.9–16.6%) of nymphs, and 20.4% (110/539; 95% Cl 17.2–24.0%) of adult *Ixodes* ticks (females: 20.6% [105/510; 95% Cl 17.3–24.3%]; males: 17.26% [5/29; 95% Cl 6.9–33.7%]). Interestingly, the *Borrelia* prevalence in ticks decreased significantly along with the duration of nymphs and females attachment from 21.6% to 11.9% (*χ*^2^_2_ = 16.5; *p* = 0.0001) (Table 1).

### Incidence of Lyme borreliosis following *Ixodes ricinus* tick bite

The clinical and diagnostic data on the incidence of LB were available for 1757 patients whose tick(s) was (were) examined for *B. burgdorferi* s.l. presence. Among them, 287 (16.3%) were bitten by infected *I. ricinus* ticks. The overall risk of developing LB after *I. ricinus* tick bite was 3.1% (95% Cl 2.3–3.9%)—the symptomatic infections were confirmed by the physicians in 54 out of 1757 individuals (Supplementary File 1). The risk of infection increased with the detection of *B. burgdorferi* s.l. DNA in ticks from 16.0% (47/287; 95%CI 12.1–20.6%) versus 0.5% (7/1470; 95% CI 0.2–0.9%) when ticks tested negative (*χ*^2^_1_ = 134.0; *p* = 0.0001). The risk of developing LB was higher after the nymphal bite (3.4%; 95% Cl 2.5–4.5%) compared with the bite by an adult tick (2.6%; 95% Cl 1.4–4.3%), however, the differences were not statistically significant (*p* = 0.088). No LB incidence was noted following a bite from a larval or male tick. The incidence of LB was similar among different age groups and ranged from 2.5% in elderly participants (> 65 years) to 3.0% in young (18–35 years) and mature (36–65 years) participants (*p* = 0.977) (Table 2). LB was diagnosed more frequently in patients who removed the tick after 48 h (5.6%) compared with those who removed it within the first 24 h of attachment (2.8%) (Table 1). However, this difference was not statistically significant (*p* = 0.125), indicating no association between the duration of tick attachment and the incidence of LB. Among *Borrelia*-infected tick bites (*n* = 250), longer feeding duration was associated with an increased risk of LB, ranging from 10.0% (95% CI 5.3–17.0%) for ticks removed within the first 24 h of attachment to 30.0% (95% CI 18.7–43.6%) for ticks removed after 48 h (*χ*^2^_2_ = 9.14; *p* = 0.010, Table 1).

**Table 2:** The frequency of the incidence of LM and EM, among participants from different age groups

	Number of participants	Age [years]^*^				*P* value
		< 18	18–35	36–65	> 65	
Incidence of LB after *I. ricinus* tick bite	1757	2.7 [1.1–5.4]	3.0 [1.7–5.1]	3.0 [2.0–4.2]	2.5 [0.8–5.7]	0.977
Incidence of EM after *I. ricinus* tick bite	1757	1.3 [0.4–3.5]	2.0 [1.0–3.8]	2.0 [1.3–3.1]	1.8 [0.5–4.8]	0.914

^*^Values are given as the percentage with the 95% confidence interval (Cl) in parentheses

EM was observed in 1.9% (34/1757; 95% Cl 1.4–2.7%) of participants who were bitten by *I. ricinus* tick, in 10.1% (29/287; 95% Cl 7.0–14.0%) of participants who were bitten by *B. burgdorferi* s.l.*-*infected tick(s) and in 64.9% (34/54; 95% Cl 50.8–76%) of participants in whom LB was diagnosed. The incidence of EM was similar in all age groups and varied from 1.3% to 2.0% (*p* = 0.914; Table 2). The EM was observed more frequently after 48 h of tick attachment (3.8%) compared with within the first 24 h (1.3%) or between 24 and 48 h (2.4%). These differences were not statistically significant (*p* = 0.075; Table 1), indicating—similarly to the incidence of LB—no clear association between the duration of tick attachment and the incidence of EM. However, among *Borrelia*-infected tick bites (*n* = 250), longer feeding duration—similarly to the incidence of LB—was associated with an increased risk of EM, ranging from 5.0% (95% CI 1.9–10.6%) for ticks removed within the first 24 h of attachment to 22.0% (95% CI 12.3–34.8%) for ticks removed after 48 h (*χ*^2^_2_ = 9.56; *p* = 0.008, Table 1).

Species differentiation of *Borrelia-*infected ticks removed from the skin of the patients with LB was successful in 40 out of 46 positive tick samples (87.0%). The most frequently detected *Borrelia* species was *B. afzelii* (27/40; 67.5%), followed by *B. garinii* (7/40; 17.5%), *B. burgdorferi* s.s. (2/40; 5.0%), *B. lusitaniae* (2/40; 5.0%) and *B. valaisiana* (1/40; 2.5%). In one tick (2.5%) the co-infection with *B. burgdorferi* and *B. miyamotoi* was detected (Supplementary File 1).

### *Borrelia miyamotoi *in *Ixodes ricinus* ticks

*Borrelia miyamotoi* infections were identified in eight *I. ricinus* ticks: in five nymphs and three adults.

### *Borrelia* load in ticks

Quantitative data reflecting *Borrelia* targets within ticks were compared separately for the ticks infected *B. burgdorferi* s.l. species complex or *B. miyamotoi*. The ticks with co-infections or the ticks infected with unknown *Borrelia* genospecies were excluded from further analysis.

The mean number of *B. burgdorferi* s.l. 16S rRNA copies detected in the ticks decreased significantly along with the time of tick attachment to the skin from 91.0 in the first 24 h (95% Cl 25.8–155.2) to 26.8 (95% Cl 0.0–66.9) after 48 h (*p* = 0.0001; Kruskal–Wallis test) (Table 3). For the ticks removed from the skin of patients with LB, the mean number of *B. burgdorferi* s.l. 16S rRNA copies (44.5; 95% Cl 0.2–94.6) were significantly lower than for the ticks collected from the skin of individuals in whom LB was not diagnosed (67.2; 95% Cl 32.9–101.6) (*p* = 0.008; Mann–Whitney test). The mean number of *B. burgdorferi* s.l. 16S rRNA copies were lower in the nymphs (60.4; 95% Cl 34.8–86.0) than in the females (71.6; 95% Cl 0.0–146.75), however, the difference was not statistically significant (*p* = 0.510; Mann–Whitney test).

**Table 3:** Load of *B. burgdorferi* s.l. in *Ixodes ricinus* ticks removed from human skin

	Number of tested ticks	Mean number of *Borrelia* 16S rRNA copies per µL of the final 1xddPCR reaction (95% confidence interval)	*P* value
Tick life stage			
Larvae	0	nd	
Nymphs	164	60.4 [34.8–86.0]	0.510
Adults	80	71.6 [0.0–146.7]	
Ʃ	244		
Time of tick attachment			
< 24 h	95	91.0 [25.8–155.2]]	0.0001
24–48 h	93	44.6 [27.0–62.2]	
> 48 h	37	26.8 [0.0–66.9]	
Ʃ	225		
*Borrelia*-infected ticks			
After biting LB was diagnosed	39	44.5 [0.2–94.6]	0.008
After biting LB was not diagnosed	205	67.2 [32.9–101.6]	
Ʃ	244		

nd: no data

The mean number of *Borrelia* 16S rRNA copies was four times higher in the ticks infected with *B. miyamotoi* (258.6; 95% Cl 51.9–465.2) than in *B. burgdorferi* s.l.- infected ticks (63.9; 95% Cl 34.3–93.5) (*p* = 0.007; Mann–Whitney test).

## Discussion

### Incidence of LB and EM after *Ixodes ricinus* tick bite

We observed an overall 3.1% risk of LB after a tick bite, which aligns with previous risk estimates of less than 5% reported in other European countries, including Sweden, Finland, Switzerland, the Netherlands, and Germany [14–16, 19–21]. Most European studies are based on a combination of seroconversion monitoring following a tick bite and clinical observation of LB symptoms, with seroconversion often considered sufficient to confirm *Borrelia* infection [15, 19]. However, in our study, only LB cases confirmed by physicians, following the recommendations of the Polish Society of Epidemiologists and Infectious Disease Physicians [26], were analyzed. This approach provides a more accurate assessment of the actual risk of developing LB, as seroconversion without clinical symptoms appears to be common in Europe and may explain the relatively high levels of seropositivity in populations from endemic areas and those at risk.

A higher proportion of LB diagnoses was observed among individuals who removed ticks after more than 48 h of attachment, compared with those who did so within the first 24 h. However, due to the relatively small number of confirmed LB cases (*n* = 54; 13 occurred after < 24 h, 22 after 24–48 h, and 19 after > 48 h), these non-significant findings should be interpreted with caution, as they may reflect limited statistical power. At the same time, we demonstrated that among tick bites involving only *Borrelia*-infected ticks, a longer feeding duration appeared to be associated with an increased likelihood of LB development. Notably, previous study has reported cases of *Borrelia* transmission and subsequent development of LB following tick attachment periods of less than 24 h [18]. Therefore, prompt removal of ticks remains a critical factor in reducing the risk of *Borrelia* spirochete transmission.

Among all our participants, 1.9% developed a typical migrating rash and 76% of the ticks removed from the skin of patients with EM were infected with *B. afzelii*. In Poland, the most common manifestations of LB are EM and LA, regardless of age, which may be attributed to this predominant *Borrelia* species in *I. ricinus* ticks [4, 31]. In our study, EM developed in nearly 65% of participants diagnosed with LB. Diagnosis based on the presence of EM is usually straightforward and leads to antibiotic treatment. In cases where EM is absent, diagnosing LB can be challenging, especially considering that up to two-thirds of tick bites in humans may go unnoticed [32]. EM appears to be more common in children than in adults [33]. Nevertheless, in our study, its incidence was similar across all age groups. Among tick bites involving *Borrelia*-infected ticks, longer feeding duration appeared to be associated with an increased likelihood of EM development, with EM most frequently observed in participants who removed the tick after more than 48 h of attachment. However, this association was not confirmed when analyzing the full dataset including both *Borrelia*-infected and non-infected ticks. Given the relatively small number of EM cases (*n* = 29), these non-significant findings should be interpreted with caution, as they may reflect again limited statistical power.

The detection of *Borrelia* DNA in a tick was one of the most significant factors associated with an increased risk of developing LB. However, in our study, LB cases were also observed following bites from *Borrelia*-negative ticks. This could be owing to other unnoticed tick bites, a *Borrelia* load in the tick below the detection limit of qPCR, or the gradual injection of the major bacterial load over the course of the tick’s attachment. This was also demonstrated in our study, where a significant decrease in *Borrelia* prevalence and load in ticks was observed as the duration of their attachment to the skin increased. Therefore, it is worth highlighting that a bite from a *Borrelia*-negative tick does not completely exclude the possibility of developing LB. In cases of LB following a *Borrelia*-negative tick bite, it is also necessary to consider the possibility of misdiagnosing skin lesions following a tick bite or infection with other tick-borne pathogens as EM in the course of LB, as well as the possibility that a tick bite occurred before the participants joined our study.

### *Borrelia *spirochetes in *Ixodes ricinus* ticks

The prevalence of *Borrelia* spirochete infection (15.7%) in *I. ricinus* ticks collected from participants was significantly lower than what we reported in similar studies conducted between 2016 and 2019 (25.3%; [10]) and relatively close to the infection rate observed in *I. ricinus* questing ticks in Poland (11.3%; [34]). A significant decrease in *Borrelia* prevalence between 2021 and 2022 in ticks collected from human skin was also observed, similar to our previous study [10]. At the same time, the number of LB cases in Poland increased slightly from 12,500 in 2021 to 17,338 in 2022 and then rose significantly in subsequent years, reaching 29,347 in 2024 [3]. In 2021 and 2022, the impact of the pandemic on the epidemiological situation of Lyme disease was still noticeable [35, 36]. During the severe acute respiratory syndrome coronavirus 2 (SARS-CoV-2) outbreak, healthcare systems postponed and scaled down certain aspects of routine medical care to minimize unnecessary hospital visits, reduce the burden on healthcare facilities, and lower the risk of infection. As a result, the availability of medical consultations for reasons other than SARS-CoV-2 infections was limited. Additionally, restrictions on outdoor activities significantly reduced the risk of tick bites. However, the decline in *Borrelia* prevalence observed in our long-term studies (2016–2019 [10]; 2021–2022, this study) does not explain the increase and relatively high incidence of LB in Poland. Czupryna et al. [7] noted that the reported incidence of LB in Poland does not correlate with trends in other tick-borne diseases (e.g., TBE), which suggests that LB may be subject to overreporting and overdiagnosis, highlighting the need for verification of the LB reporting system in Poland.

Our results align with other European surveys, where *Borrelia* prevalence in ticks removed from humans ranges from 6% in Italy to as high as 30% in the Netherlands [17, 18, 37–42]. In agreement with previous reports [10, 22, 34, 38, 39, 43], adult ticks were more often infected with *Borrelia* than nymphs, likely due to the higher number of blood meals ingested by adult ticks. As mentioned above, we examined how *Borrelia* prevalence and the load of *Borrelia* spirochetes changed depending on the tick life stage and the duration of tick attachment. Our results have indicated that the risk of developing LB is higher after a nymphal bite than after an adult tick bite. Nevertheless, this may be due to the small size of nymphs, which often go unnoticed while feeding on human skin. This trend was also observed in our study, where nymphs were predominantly removed after 24 h of attachment, whereas adult females were mostly collected within the first 24 h.

No statistically significant differences in *Borrelia* load were observed between nymphs and females. However, *Borrelia* prevalence and the number of spirochetes decreased significantly with the duration of tick attachment, which may reflect the transmission of these bacteria from the tick to the human host. A similar phenomenon was observed previously [18, 22]. To confirm that this process results from transmission, further studies are needed to investigate changes in *Borrelia* cell numbers in ticks during feeding on the host’s skin.

In our study, the *Borrelia* load in ticks collected from the skin of patients diagnosed with LB was significantly lower than in ticks collected from individuals who did not develop the disease. Although we were unable to assess the *Borrelia* load in ticks prior to or during feeding, our findings suggest that both the *Borrelia* load within the tick and the efficiency of *Borrelia* transmission from tick to host may influence the development of LB. It has been speculated that ticks harboring a higher *Borrelia* load may pose a greater risk of transmission. Wilhelmsson et al. [20] reported no statistically significant difference in *Borrelia* load between ticks removed from participants who later seroconverted and those who did not. However, participants who seroconverted had removed their *Borrelia*-infected ticks significantly later than those who did not seroconvert. Despite these observations, the mechanisms and factors influencing *Borrelia* transmission efficiency remain poorly understood.

### *Borrelia miyamotoi* in *Ixodes ricinus* ticks

Surprisingly, in our study, the prevalence of *B. miyamotoi* was significantly lower than we observed previously (3% versus 15.8%; [10]). However, it remains comparable to reports from other European studies, where prevalence usually does not exceed 5% [22, 44]. Notably, the transmission rate of *B. miyamotoi* from tick to human is estimated at 8.3%, twice that of *B. burgdorferi* s.l. [45]. We have observed that the mean number of *Borrelia* 16S rRNA copies was four times higher in *B. miyamotoi-*infected ticks than in *B. burgdorferi* s.l.-infected ticks. In our study, the frequency of co-infections with different *Borrelia* species in ticks removed from the skin of LB-diagnosed patients was extremely low (2.5%) suggesting that the risk of *B. miyamotoi/B. burgdorferi* s.l. co-infection in humans is negligible. Standard LB serologic tests may not detect BMD, and current BMD serology relies on the glpQ gene, which is absent in LB spirochetes [46, 47]. Although PCR is recommended for diagnosing acute BMD, its sensitivity is limited, and false negatives are possible [48]. Owing to these diagnostic limitations, BMD cases may be underreported.

Some limitations in our study should be also considered. Since the ticks were sent by mail or via a delivery company, the pre-analytical process (i.e., tick storage after removal from the skin) was beyond our control and may have affected the molecular analysis, including pathogen detection and genotyping. Additionally, *Borrelia* detection in ticks was based on DNA fragments of the bacterial genome, without considering spirochete viability or infectivity. The second set of questionnaires was collected 8 weeks after the tick bite, meaning that late LB manifestations remained unknown. It is important to note that the estimation of tick attachment time was based on studies conducted using ticks engorged on laboratory animals (rabbits), not on humans. Furthermore, in our study, ticks were collected from multiple body regions, which could potentially affect the rate of engorgement.

## Conclusions

In Poland, the overall risk of LB following an *I. ricinus* tick bite is relatively low (3.1%). However, among *Borrelia*-infected ticks, the risk increases substantially with the duration of tick attachment, reaching up to 30% after 48 h. The results of this study indicate that EM developed in two-thirds of participants diagnosed with LB. Further studies are needed to confirm any potential correlation between the risk of developing LB and the *Borrelia* load in ticks as well as to investigate the dynamics of *Borrelia* loads before, during, and after blood-feeding. Although *B. afzelii* is the predominant species identified in *I. ricinus* ticks, the risk of *B. miyamotoi* infection following a tick bite should also be considered, given the significantly higher *B. miyamotoi* loads in ticks, the occurrence of co-infections and their potential influence on infection dynamics, and the tick vector as well as the endemic regions for LB and BMD overlap. Our findings could be valuable for assessing an individual’s risk of infection after a tick bite and for developing a well-founded prophylactic strategy. We also emphasize that citizen science, despite its limitations, is an effective method for collecting data on tick bites in humans, enabling the assessment of exposure risk to tick-borne pathogens and supporting epidemiological surveillance.

## Supplementary Information

Supplementary file 1: Participants diagnosed with Lyme Borreliosis after *I. ricinus* tick bite during two years of study.

## Data Availability

The datasets used and analyzed during this study are available from the corresponding author (RWF) upon a reasonable request. The new nucleotide sequences of *Borrelia* species have been deposited in the GenBank database under accession numbers: PV173335–PV173337, PV173339–PV173341.

## Abbreviations

LB: Lyme borreliosis
EM: *Erythema migrans*
RFLP: Restriction fragment length polymorphism
ddPCR: Droplet digital PCR
