# Supplementary material for: Incidence of Lyme borreliosis following Ixodes ricinus tick bites in Poland: a citizen science approach
Source: Parasit Vectors. 2025 Nov 28;18:494. doi: 10.1186/s13071-025-07133-y (PMC12664187; doi:10.1186/s13071-025-07133-y)
Supplement: Supplementary file 1 — Participants diagnosed with Lyme Borreliosis after I. ricinus tick bite during two years of study. [file 13071_2025_7133_MOESM1_ESM.docx]

**Supplementary File 1.** Participants diagnosed with Lyme Borreliosis after *Ixodes ricinus* tick bite during two years of study (2021-2022)

| **No.** | **Sex** | **Age** | **I. ricinus stage^a^** | **Time of feeding (h)** | **Borrelia species detected in I. ricinus tick** | **Erythema migrans presence** | **Serological test ordered by GP to confirm LB ^b^** | **Result of serological tests** | **Antibiotic treatment ordered by GP** |
| --- | --- | --- | --- | --- | --- | --- | --- | --- | --- |
| 1 | F | 62 | F | >48 | *B. lusiataniae* | No | Yes | Positive | Yes |
| 2 | F | 41 | N | <24 | Tick negative | Yes | No | - | Yes |
| 3 | M | 46 | F | <24 | *B. burgdorferi/B. miyamotoi* | No | Yes | Positive | Yes |
| 4 | M | nd | N | >48 | *B. afzelii* | No | Yes | Positive | Yes |
| 5 | M | 56 | N | 24-48 | *B. garinii* | Yes | No | - | Yes |
| 6 | F | 45 | N | 24-48 | *B. afzelii* | Yes | No | - | Yes |
| 7 | F | 41 | N | >48 | *B. afzelii* | Yes | No | - | Yes |
| 8 | F | 30 | N | >48 | *B. garinii* | No | Yes | Positive | Yes |
| 9 | F | 22 | N | >48 | *B. afzelii* | No | Yes | Positive | Yes |
| 10 | F | nd | N | <24 | *B. garinii* | No | Yes | Positive | Yes |
| 11 | M | 37 | N | 24-48 | *B. afzelii* | No | Yes | Positive | Yes |
| 12 | F | 39 | N | 24-48 | *B. afzelii* | Yes | No | - | Yes |
| 13 | F | 47 | N | >48 | *B. afzelii* | No | Yes | Positive | Yes |
| 14 | F | 73 | N | 24-48 | *B. afzelii* | Yes | No | - | Yes |
| 15 | F | 64 | N | 24-48 | *B. garinii* | No | Yes | Positive | Yes |
| 16 | M | 26 | N | 24-48 | *B. valaisiana* | No | Yes | Positive | Yes |
| 17 | F | 56 | N | 24-48 | *B. afzelii* | No | Yes | Positive | Yes |
| 18 | F | 44 | N | <24 | nd | No | Yes | Positive | Yes |
| 20 | M | 31 | N | 24-48 | *B. afzelii* | No | Yes | Positive | Yes |
| 21 | F | 38 | N | 24-48 | Tick negative | Yes | Yes | Negative | Yes |
| 22 | M | 38 | N | <24 | *B. garinii* | Yes | Yes | Positive | Yes |
| 23 | M | 32 | N | >48 | *B. afzelii* | Yes | No | - | Yes |
| 24 | F | 73 | N | >48 | *B. afzelii* | Yes | Yes | Positive | Yes |
| 25 | F | 21 | F | 24-48 | *B. afzelii* | Yes | No | - | Yes |
| 26 | F | 65 | F | >48 | *B. burgdorferi* | Yes | No | - | Yes |
| 27 | M | 40 | F | >48 | *B. afzelii* | Yes | Yes | Negative | Yes |
| 28 | M | 39 | N | >48 | *B. garinii* | Yes | No | - | Yes |
| 29 | M | 34 | N | nd | *B. afzelii* | Yes | No | - | Yes |
| 30 | M | 47 | F | <24 | Tick negative | No | Yes | Positive | Yes |
| 31 | F | 46 | N | nd | Tick negative | Yes | No | - | Yes |
| 32 | F | 10 | N | 24-48 | *B. afzelii* | Yes | No | - | Yes |
| 33 | M | 72 | F | 24-48 | *B. burgdorferi* | No | Yes | Positive | Yes |
| 34 | F | 37 | F | 24-48 | Tick negative | Yes | Yes | Positive | Yes |
| 35 | M | 60 | F | >48 | *B. afzelii* | Yes | No | - | Yes |
| 36 | M | 69 | N | <24 | *B. afzelii* | Yes | No | - | Yes |
| 37 | F | 15 | F | 24-48 | nd | No | Yes | Positive | Yes |
| 38 | M | 34 | N | <24 | *B. afzelii* | Yes | No | - | Yes |
| 39 | M | 19 | N | 24-48 | *B. garinii* | Yes | No | - | Yes |
| 40 | F | 32 | N | >48 | *B. afzelii* | Yes | No | - | Yes |
| 41 | M | 10 | N | <24 | *B. afzelii* | No | Yes | Positive | Yes |
| 42 | F | 31 | N | nd | *B. afzelii* | Yes | No | - | Yes |
| 43 | F | 53 | N | 24-48 | *B. afzelii* | Yes | Yes | Negative | Yes |
| 44 | F | 37 | N | 24-48 | nd | Yes | No | - | Yes |
| 45 | F | 47 | N | <24 | *B. lusitaniae* | Yes | Yes | Negative | Yes |
| 46 | F | 60 | N | 24-48 | Tick negative | Yes | No | - | Yes |
| 47 | F | 10 | N | >48 | *B. afzelii* | Yes | Yes | Positive | Yes |
| 48 | F | 55 | F | >48 | Tick negative | No | Yes | Positive | Yes |
| 49 | F | 48 | F | 24-48 | nd | Yes | No | - | Yes |
| 50 | M | 33 | N | 24-48 | nd | Yes | No | - | Yes |
| 51 | F | nd | N | <24 | nd | Yes | No | - | Yes |
| 52 | M | 42 | N | <24 | *B. afzelii* | No | Yes | Positive | Yes |
| 53 | F | 36 | N | 24-48 | *B. afzelii* | Yes | Yes | Positive | Yes |
| 54 | F | 7 | N | 24-48 | *B. afzelii* | Yes | no | - | Yes |

^a^ N- nymph, F- female

^b^ Serological tests confirmed LB were based on a two-tiered algorithm (ELISA and Western Blot tests) according to the recommendations of The Polish Society of Epidemiology and Infectious Disease (Moniuszko-Malinowska et al. 2023) and European guidelines (Stanek et al. 2011). Yes- test/tests were ordered by GP; No- test/tests were not ordered by GP.

nd- no data
